# Microvascular Reconstruction of Iatrogenic Femoral Artery Thrombosis in an Infant: A Case Report and Review of the Literature: Infant Femoral Artery Reconstruction

**Published:** 2009-06-02

**Authors:** Michael J. Salvino, Raja Ramaswamy, Loren S. Schechter

**Affiliations:** ^a^ Loyola University Medical Center, Maywood, IL; ^b^ Advocate Lutheran General Hospital, Park Ridge, IL

## Abstract

**Objective:** We present a case of femoral artery thrombosis and acute limb ischemia in an infant following attempted femoral venipuncture. Microsurgical reconstruction of the lesion and a review of the literature are described. **Methods:** Insertion of intravascular catheters is a commonly performed procedure in neonatal and pediatric intensive care units. However, significant iatrogenic injuries to the peripheral vascular system can occur. Management ranges from anticoagulation and thrombolytics to surgical intervention. In this case, surgical therapy involved thrombectomy and microsurgical reconstruction of the femoral artery. **Results:** Vascular inflow was reestablished and the limb was salvaged. **Conclusions:** We believe that microsurgical techniques are an important component for successful vascular reconstruction after iatrogenic artery thrombosis in an infant. Microvascular techniques allow for a controlled operating field with magnification, thereby assisting in the accuracy of vessel repair.

Insertion of intravascular catheters is a commonly performed procedure in neonatal intensive care units (NICUs). Most critically ill newborns will require an intravascular catheter for therapies ranging from blood pressure monitoring to intravenous infusions and nutritional support. Significant iatrogenic injuries to the peripheral vascular system can occur. Management of these injuries ranges from the use of thrombolytics and anticoagulation to surgical intervention. We present a case of femoral artery thrombosis and acute limb ischemia in an infant following attempted femoral venipuncture. Microsurgical reconstruction of the lesion and a review of the literature are described.

## CASE EXAMPLE AND DESCRIPTION OF SURGICAL TECHNIQUE

A female triplet, born at a gestational age of 31 weeks, weighing 3.5 kg was hospitalized in the NICU for 2 weeks following delivery. Four weeks after discharge, she was readmitted with presumptive sepsis with decreased oral intake, tachycardia, and fussiness. Her weight had decreased to 3.2 kg.

Multiple unsuccessful attempts at femoral venous cannulation were made. A follow-up lower extremity duplex examination was performed and revealed occlusion of the distal right external iliac artery and common femoral artery. A pulse oximeter on the toes of the affected extremity revealed no waveform. A subsequent magnetic resonance angiogram confirmed the arterial occlusion. The patient was managed medically with systemic heparinization. After approximately 96 hours, the extremity was cool, and the patient exhibited decreased movement of the foot and ankle. Vascular and plastic surgery services were consulted. The patient was taken to the operating room following the worsening clinical condition and lack of improvement with 96 hours of medical therapy.

Through a 2-cm incision centered over the femoral artery, the common femoral artery and vein were identified. The common femoral vein was noted to be patent by intraoperative Doppler examination. However, the common femoral artery was occluded and distal Doppler signals were not present. The artery was isolated for a distance of 5 mm, obtaining proximal and distal control. A transverse arteriotomy was performed and a 2-French Fogarty catheter was inserted approximately 10 cm proximally and distally. A small amount of fibrinoid clot was returned and intraluminal irrigation of the vessel was performed with heparin. Pulsatile flow was established and the arteriotomy was closed under the operating microscope with interrupted 9-0 Prolene. Doppler examination revealed an arterial signal distal to the arteriotomy with signals now present in the distal extremity. The external diameter of the vessel was slightly less than 1 mm, and the internal diameter was approximately 600 microns. Following revascularization, palpation of the calf revealed a tense anterior compartment. A prophylactic 4-compartment fasciotomy was performed through medial and lateral incisions on the leg. The patient was placed on a heparin drip postoperatively, and the partial thromboplastin time (PTT) was maintained at a goal of 65. Serial cranial ultrasounds were performed to evaluate for intracranial hemorrhage. On the first postoperative day, the patient developed a groin hematoma. In addition, the foot had become acutely swollen and appeared ischemic. The patient was returned to the operating room for evacuation of the groin hematoma and a fasciotomy of the foot. Groin exploration revealed bleeding from the femoral suture line. This was repaired with a single 10-0 nylon suture. The fasciotomy of the foot was performed through incisions over the dorsalis pedis and posterior tibial arteries. Upon release of the extensor retinaculum in the foot, a Doppler signal returned in the posterior tibial artery.

All skin incisions on the foot and leg except the lateral leg fasciotomy were sutured. The open wound on the leg was managed with negative pressure therapy at 25 mmHg. Monitoring of foot perfusion was performed with pulse oximetry placed on the great toe. Pulse oximetry readings were maintained over 95% throughout the postoperative period. Clinically, the leg remained pink, warm, and well-perfused. The patient moved the lower leg spontaneously but did not appear to move the foot and toes.

**Figure 1:**
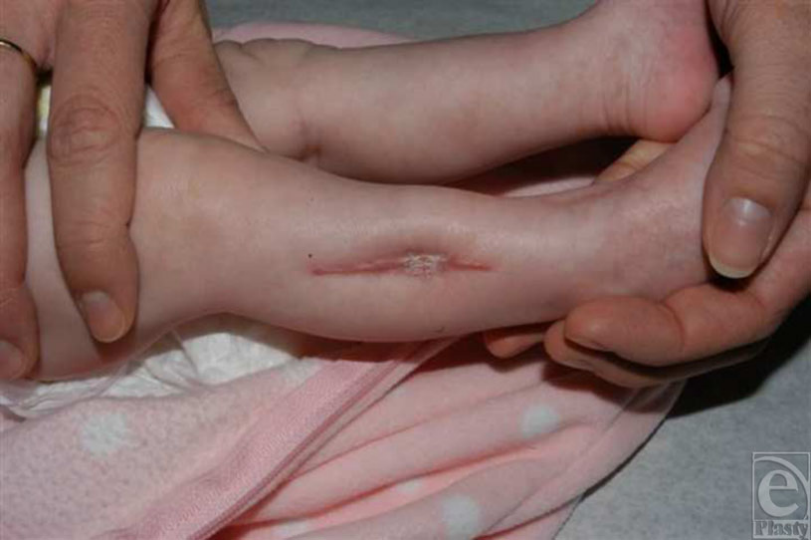
Healed fasciotomy site.

**Figure 2:**
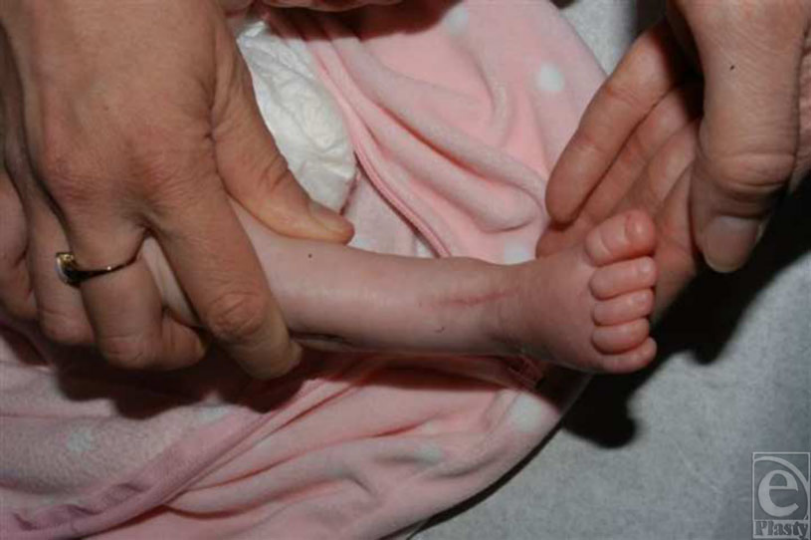
Salvaged limb.

At 1-month follow-up, the patient demonstrated improved function of the extremity, as evidenced by spontaneous foot and toe movement. Doppler examination at this time revealed triphasic signals in the femoral artery. In addition, the lateral leg healed to completion by secondary intention (Figs 1 and 2).

## LITERATURE REVIEW

Only 2 previous case reports describe microvascular reconstruction of iatrogenic vascular injuries in infants. Most cases of surgical repair focus on the pediatric population and children older than 2 years. Classic surgical procedures include arterial thrombectomy, fem-fem bypass, femoral patch angioplasty, and femoral thrombectomy with repair.^1^,^2^

The first report involving microvascular techniques to reconstruct iatrogenic vascular injuries in neonates and small children was described by LaQuaglia et al^3^ in 1991. The median age at repair was 28 days. In their series of 5 patients with femoral artery thrombosis, microvascular reconstructive techniques led to successful arterial reconstruction and limb salvage. However, one patient died from complications unrelated to the surgery.^3^

Friedman et al^4^ used primary thrombectomy with microvascular techniques in 2 neonates with thrombosis of the femoral and brachial arteries, respectively. The brachial artery repair remained patent but the femoral artery thrombosed after 18-month follow-up. The authors note that although the femoral artery thrombosed, there was no leg-length discrepancy.^4^

In a case series of 6 neonates with extremely low or low birth weight, Gamba et al^5^ describe the microsurgical repair of AV fistulas and carotid lesions. This case series also highlighted 2 cases of limb ischemia treated with primary thrombolytic therapy. One patient underwent amputation at the knee level.^5^

## DISCUSSION

Most newborns in the NICU will receive some form of intravascular catheter. As such, an increase in iatrogenic vascular lesions has been observed.^5^ The most common complications associated with catheter placement include hemorrhage, arteriovenous fistula formation, infection, vascular spasm, and thrombosis with subsequent thromboembolic events.^6^ In severe cases, thrombosis can lead to acute or chronic limb ischemia, resulting in limb length discrepancy, gait disturbance, or gangrene.^1^ In a retrospective study by Lin et al,^1^ ischemic complications were reported in 21 of 1674 patients over a 5-year period.

The femoral artery is the most preferred access site for procedures in the NICU.^1^ This is associated with an increased risk of groin complications and septic arthritis of the hip.^7^ Furthermore, because the femoral artery is an end artery, thromboembolic complications can lead to limb ischemia. In contrast to adults with iatrogenic femoral injuries, the pediatric population presents special challenges due to the small vessel caliber and increased risk for thrombosis due to undeveloped coagulation mechanisms. Heparin, when used as prophylaxis, has been shown to reduce the incidence of vessel occlusion in neonates. However, heparin carries the risk of intracranial hemorrhage, allergic reactions, and heparin-induced thrombocytopenia.^8^ Besides that, recognition of limb ischemia in neonates may be delayed because of the inability to communicate. While vascular obstruction may present with pulselessness, other, more subtle signs such as decreased skin temperature, skin discoloration, and decreased range of motion may be indicative of impaired circulation.^3^,^4^ Though rare, the process of catheter placement in neonates can lead to serious sequelae, including acute limb ischemia. If not promptly treated, this can lead to gangrene or long-term sequelae such as limb length discrepancies and gait disturbance. Most commonly these events are iatrogenic.^1^

Acute limb ischemia can be confirmed with a Doppler study. However, in patients with a high pretest probability of a thrombus, a negative study does not preclude the presence of a thrombus. As in our case report, physical examination may determine whether or not operative intervention is required. However, differentiating between spasm and thrombosis may be difficult. Typically, spasm resolves within 4 to 6 hours after catheter removal.^2^,^3^,^5^

Many centers opt to treat acute neonatal limb ischemia nonoperatively with anticoagulation. This may be due to the small size of the vessels and contributing factors such as the underlying disease process. Flanigan et al^2^ report successful return of normal ankle/brachial index in 93% of patients treated nonoperatively with heparin. However, these results are in patients who maintain palpable femoral pulses. As in our case, patients with absent femoral pulses should be considered for operative intervention.^2^ While nonoperative treatment may result in restoration of perfusion, chronic femoral artery occlusion (>30 days) can result in significant limb growth impairment. Flanigan et al^2^ report that 23% of nonsurgically versus 9% of surgically treated children developed leg length discrepancies (0.5–3.0 cm) as a result of ischemia lasting greater than 30 days.

In this case, thrombectomy and microsurgical reconstruction of the femoral artery were performed. In a review of the literature, we found no precedent to define who, among the surgical specialists, should be involved in neonatal revascularization. In this instance, both vascular and plastic surgery services were involved. We believe that microsurgical techniques are an important component for successful vascular reconstruction in this setting. Microvascular techniques allows for a controlled operating field with magnification, thereby assisting in the accuracy of vessel repair.

There are no sources of support or funding to disclose.
